# Management of C1-C2 Myelopathy Due to Periodontoid Pseudotumor With a Successful Posterior Stabilization and Decompression: A Case Report

**DOI:** 10.7759/cureus.89730

**Published:** 2025-08-10

**Authors:** Joana Azevedo, David Ferreira, Ana Vilela, Pedro Varanda, Bruno Santos

**Affiliations:** 1 Orthopaedics and Traumatology, Hospital de Braga, Braga, PRT

**Keywords:** atlanto-axial joint, cervical spinal cord compression (cscc), periodontoid pseudotumor, spinal-fusion, synovial pannus

## Abstract

Periodontoid pseudotumors are caused by chronic instability in the atlantoaxial joint, possibly leading to cervical myelopathy. The classic treatment is transoral resection of the pannus, followed by posterior stabilization. More recently, some cases showed regression of the inflammatory mass following stabilization alone. A 71-year-old female patient presented with cervical pain and decreasing muscular strength and dexterity in the upper limbs. Objectively, muscular strength grade IV, Hoffman sign positive bilaterally, unbalanced and wide base gait and changes in voiding pattern were noted. The CT scan showed a slight C1-C2 subluxation and C3-C6 diffuse idiopathic skeletal hyperostosis. MRI showed a large retro-odontoid inflammatory mass with stenosis at C1-C2 and multilevel discopathy with degenerative changes causing stenosis at the C5-C6 level, leading to severe C1-C2 and C5-C6 myelopathy. The patient was subjected to a C1-C6 laminectomy and occipito-C6 posterior fixation. After surgery, muscular strength, gait difficulties and pain slowly improved, with acquired autonomy. Control MRI, one year after surgery, showed complete resolution of the periodontoid pannus. In the present case, we were able to achieve a satisfactory result, with clinical improvement and radiological resolution of the inflammatory mass, with posterior stabilization of the cervical spine, without transoral approach.

## Introduction

Periodontoid pseudotumors, also referred to as synovial periodontoid pannus, are inflammatory masses resulting from chronic instability of the atlantoaxial joint. These masses typically form posterior to the odontoid process and are most commonly observed in patients diagnosed with rheumatoid arthritis. However, they can also arise in other conditions such as acute trauma, degenerative arthropathies like diffuse idiopathic skeletal hyperostosis (DISH) and ossification of the posterior longitudinal ligament (OPLL), os odontoideum, pseudoarthrosis of the odontoid process and also congenital syndromes like Down syndrome, Morquoi syndrome and neurofibromatosis [1,2]. These masses are characterized by abnormal growth of inflammatory, fibrous and granulation tissue as a result of continuous injury to the atlantoaxial joints [3]. In addition to the chronic instability, these masses can grow and exert a mass effect, leading to compression of the cervical spine and cervical nerves, which may result in severe neck pain, cervical myelopathy or radiculopathy, manifesting as sensory changes, weakness, and gait disturbances, among others. In severe cases, this compression can cause fatal outcomes due to involvement of the medulla oblongata, such as respiratory failure and even death [4,5]. The standard treatment for these conditions is transoral direct resection of the pannus and odontoidectomy, followed by posterior stabilization, thus decompressing the spinal cord [6-8]. This procedure was associated with several complications such as cerebrospinal fluid leakage and infections [3]. More recently, however, some cases have shown regression of the inflammatory mass following posterior stabilization and decompression alone, particularly in patients with rheumatoid arthritis, without the need for direct resection [8-12].

Herein, we report a case of C1-C2 myelopathy caused by compression from a periodontoid pseudotumor, complicated by multilevel discopathy resulting in C5-C6 myelopathy, and DISH affecting the C3-C6 vertebrae.

## Case presentation

**Figure 1:**
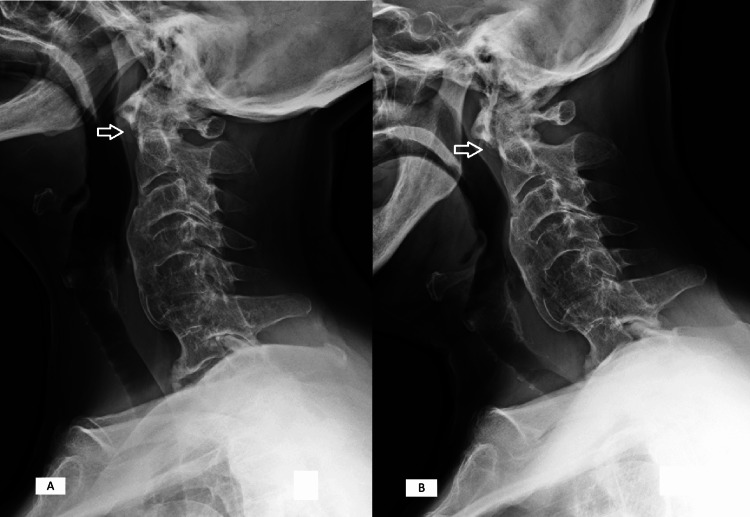
Dynamic X-rays (A) Hyperextension; (B) hyperflexion. Arrow shows slight C1-C2 subluxation, with an increase of the anterior atlanto-odontoid interval between images A and B.

**Figure 2:**
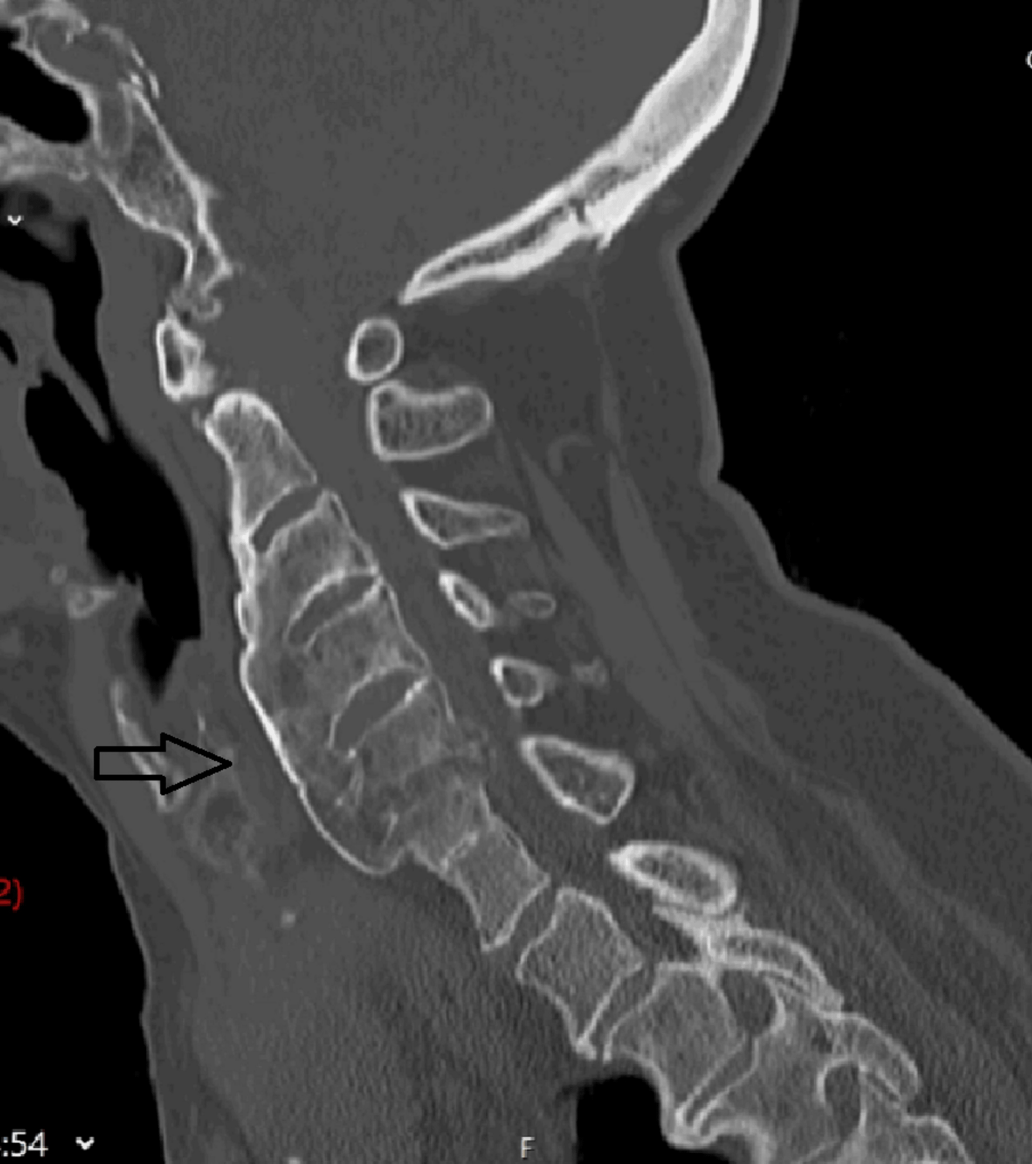
CT scan before surgery. Arrow shows C3-C6 diffuse idiopathic skeletal hyperostosis

We present a case of a 71-year-old female patient with a medical history of hypothyroidism and hyperuricemia who presented to our department due to long-standing cervical pain (Neck Disability Index scale of 33 points), with recent worsening, along with a decline in muscular strength and dexterity in the upper limbs. On physical examination, we were able to detect a decline in muscle strength with grade IV muscle strength in both the upper and lower limbs, a positive Hoffman sign bilaterally, unbalanced and wide base gait, and changes in her voiding pattern, including difficulty initiating urination. The patient's clinical presentation was classified as C in the American Spinal Injury Association (ASIA) Impairment Scale and scored nine points in the modified Japanese Orthopaedic Association (mJOA) score. The dynamic radiographies performed showed mild C1-C2 subluxation (Figure 1) and a CT scan revealed C3-C6 diffuse idiopathic skeletal hyperostosis (Figure 2).

**Figure 3:**
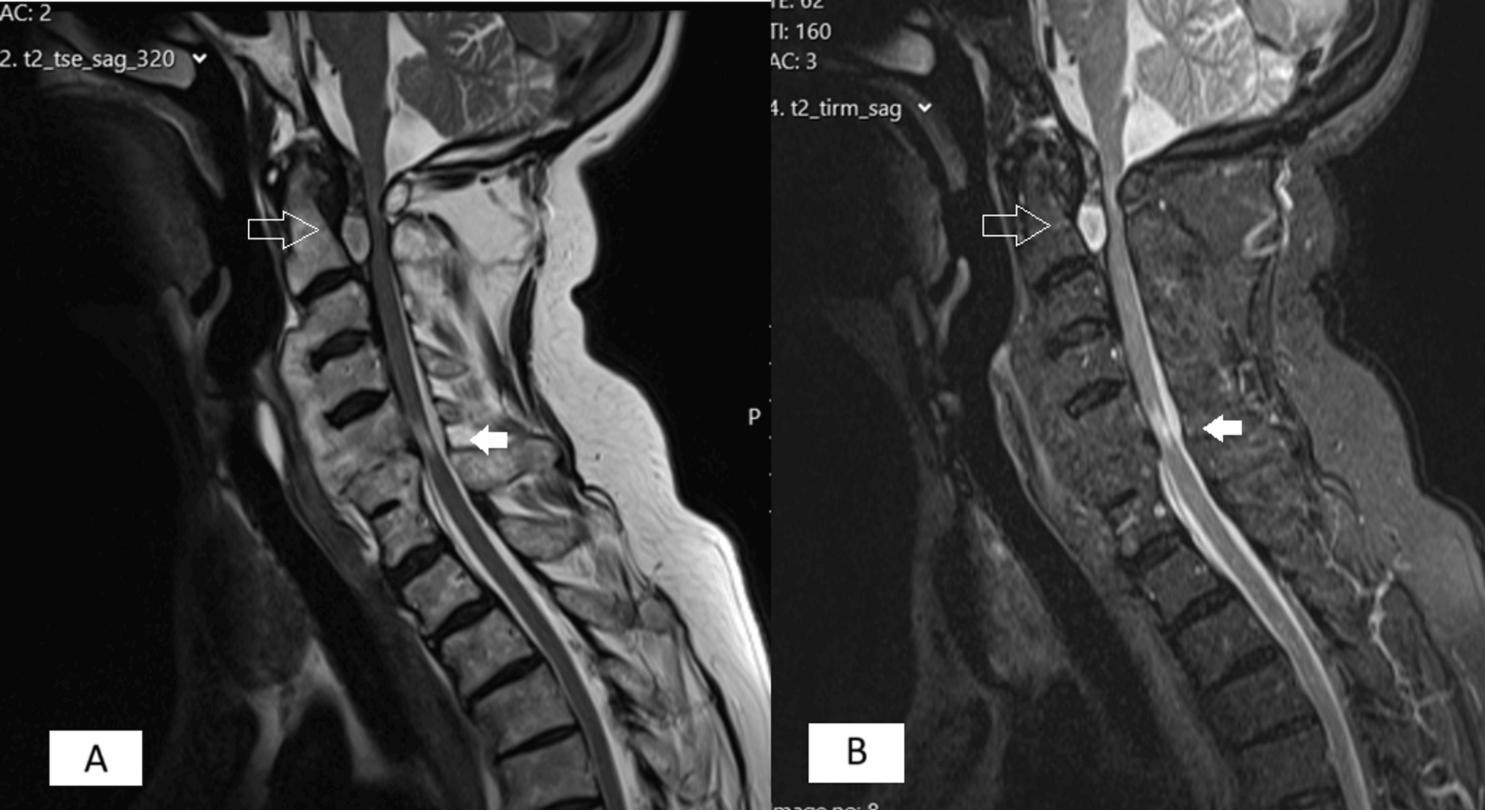
MRI before surgery Unfilled arrow shows a retro-odontoid inflammatory mass with stenosis of the vertebral canal at C1-C2 and filled arrow shows a multilevel discopathy with degenerative changes causing stenosis at C5-C6 level. (A) T2-weighted image. (B) short tau inversion recovery (STIR) image

An urgent MRI was subsequently performed, which demonstrated a large retroodontoid inflammatory mass causing stenosis of the vertebral canal at the C1-C2 level. Additionally, multilevel discopathy with degenerative changes was noted, leading to stenosis at the C5-C6 level, and contributing to severe C1-C2 and C5-C6 myelopathy (Figure 3). Rheumatoid and inflammatory markers were within the normal range.

**Figure 4:**
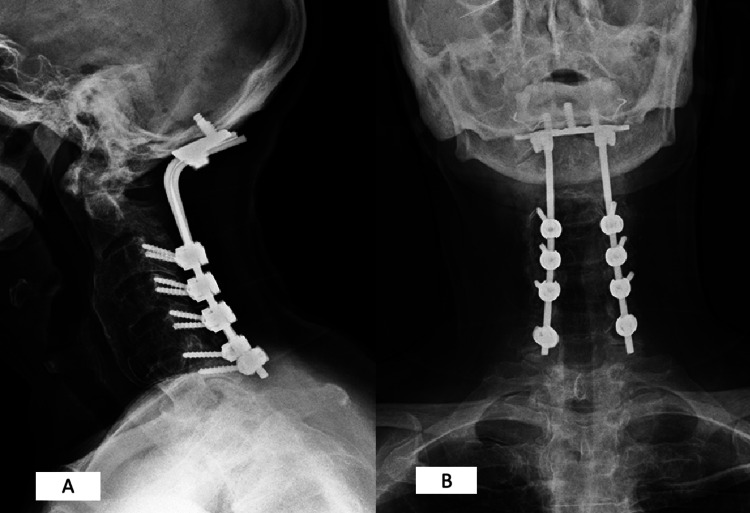
Post-operative radiography. C1-C6 laminectomy and occipito-C6 posterior fixation. (A) Lateral radiography. (B) Anteroposterior radiography

Considering the clinical and radiological features, along with the rapid progression of neurological symptoms, the patient underwent decompression and posterior stabilization after a comprehensive discussion and extensive surgical planning within four months of the beginning of symptoms. The patient was placed in a prone position, and the head was fixed using a Mayfield head holder. A C1-C6 laminectomy was performed, followed by occipito-C6 posterior fixation using occipital plates and screws for stabilization of the lateral masses (Figure 4).

**Figure 5:**
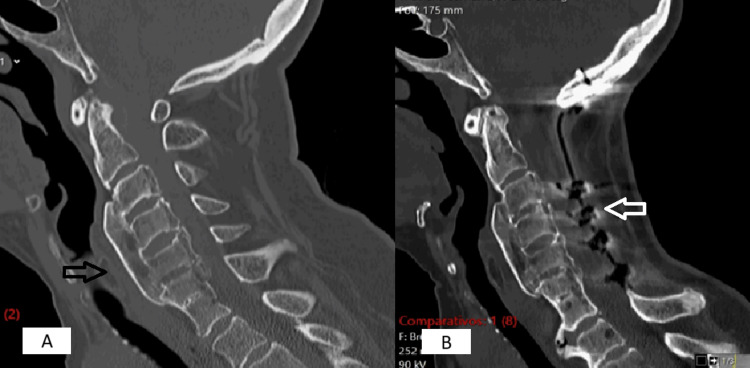
Comparison between pre- and post-operative computed tomography scans. (A) CT before surgery. The black arrow shows C3-C6 diffuse idiopathic skeletal hyperostosis. (B) CT after surgery. The white arrow shows a wide decompression of the cervical spinal cord

Postoperatively, the patient gradually experienced improvements in muscle strength, gait, and pain (Neck Disability Index scale of 14 points), with regained autonomy. She was able to walk independently, her voiding pattern normalized and muscle strength improved to grade V/V in certain segments of the upper limbs (Figure 5). The patient's clinical presentation in the last follow-up, four years after surgery, improved to a D in the ASIA Impairment Scale and scored 14 points in the mJOA score.

**Figure 6:**
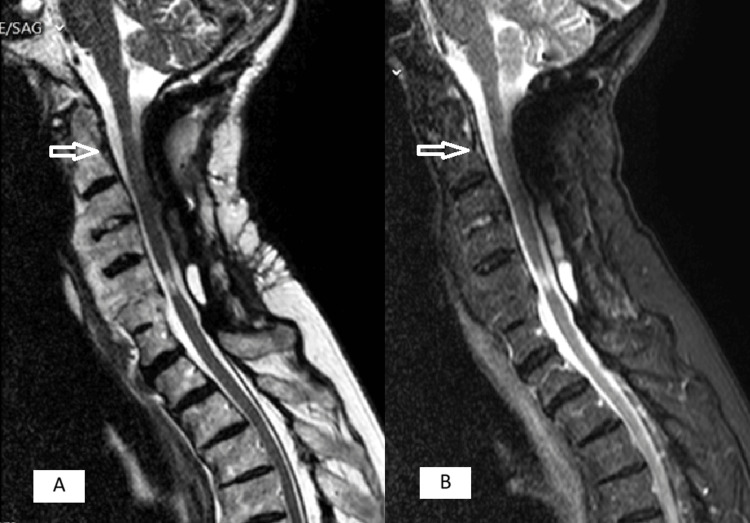
MRI after surgery Arrow shows complete resolution of the periodontoid pannus. (A) T2-weighted image. (B) Short tau inversion recovery (STIR) image

However, complete recovery was not achieved due to the established C5-C6 myelomalacia. Follow-up MRI performed one year after surgery revealed complete resolution of the periodontoid pannus (Figure 6).

## Discussion

Chronic atlantoaxial instability appears to play a crucial role in the development of synovial pannus, a condition more commonly observed in patients with inflammatory diseases such as rheumatoid arthritis and psoriatic arthritis. However, it can also occur in other causes of instability, including degenerative arthropathies and odontoid pseudoarthrosis [12].

The precise nature of the tissue remains controversial. Some authors suggest that the pannus originates from the synovium between the odontoid process and the C1 articular facets, or the transverse ligament, and consists of inflammatory granulation tissue resulting from the proinflammatory state [13]. However, since this mass is also present in patients without inflammatory diseases, other hypotheses have been proposed. Some researchers stated that the mass represents fibrous tissue, a reactive response to the persistent mechanical stress caused by instability in the atlantoaxial joint [8,13,14]. This synovial pannus or odontoid pseudotumor typically resides retroodontoid and is a significant cause of cervical pain. As it progresses, it can lead to cervical canal stenosis and, in some cases, severe myelopathy.

Although transoral odontoidectomy followed by posterior stabilization has been the classic treatment, for example, Crockard et al. in 1986 describes 14 cases of cervical myelopathy treated with transoral decompression and neurological improvement after surgery [6], several recent reports have described cases in which pannus resolution occurred with posterior stabilization alone, without the need for a transoral approach [9-13]. Lagares et al. describes a case of retroodontoid mass in the absence of rheumatoid arthritis, with pannus resolution after occipitocervical fusion [13]. Also, Young and Boyko reported three cases of periodontoid pannus formation, in the absence of inflammatory diseases, associated with chronic fractures of the dens and treated with C1/C2 transarticular screw fixation, with MRI at six months demonstrating resolution of the ventral pannus [10]. In the work presented by Joly-Torta et al., they describe two cases of periodontoid masses in middle-aged women, submitted to occipitocervical fixation, without the diagnosis of inflammatory diseases, but one had a history of dens fracture and the other had an anterior cervical arthrodesis in the past [9]. We present a case that combines C1-C2 myelopathy caused by a periodontoid pseudotumor with extensive degenerative changes in the subaxial cervical spine, leading to C5-C6 myelopathy.

Despite the complexity of the treatment, due to the multiple changes in the cervical spine and the rapid progression of symptoms, we achieved a satisfactory outcome, with clinical improvement and radiological resolution of the inflammatory mass following posterior stabilization and decompression, without the need for an anterior approach and, that way, avoiding the most common complications like dysphagia and velopharyngeal insufficiency (VPI), cerebrospinal fluid (CSF) leak and meningitis [15]. Since hypermobility of the atlantoaxial joint is considered one major cause of pannus formation, our rationale was that a rigid construct would be the best option to avoid the progression of myelopathy in the upper cervical spine and mass regression; considering that the patient had a preserved cervical lordosis and anterior spine was already anteriorly fused from C3 to C7 due to DISH (Figure 2), there were no advantages of anterior or double approach in this particular case.

## Conclusions

The favorable clinical and radiological outcomes in this case suggest that posterior stabilization and decompression of the craniovertebral junction is a safe and effective alternative to transoral pannus resection, even in patients without underlying inflammatory diseases. This approach not only avoids the significant risks associated with anterior surgery - such as dysphagia, velopharyngeal insufficiency, cerebrospinal fluid leak and meningitis - but also demonstrates excellent potential for pannus resolution. Despite the technical complexity of the procedure, we achieved substantial radiological improvement and meaningful clinical recovery at the final follow-up. These results align with growing evidence supporting posterior-only strategies in similar cases. Overall, our experience reinforces the viability of this less invasive yet highly effective surgical approach.
